# Adolescent tickling incorporating handling is associated with reduced stress- and fear-related behaviors in socially isolated male rats

**DOI:** 10.3389/fnbeh.2026.1869518

**Published:** 2026-09-03

**Authors:** Michimasa Toyoshima, Reina Tachihara, Tingbi Xiong, Keita Igarashi, Yukio Ichitani, Kazuo Yamada, Miyo Hori

**Affiliations:** 1 Institute of Human Sciences, University of Tsukuba, Tsukuba, Japan; 2 Neuroscience Degree Program, Graduate School of Comprehensive Human Sciences, University of Tsukuba, Tsukuba, Japan; 3 Faculty of Applied Psychology, Tokyo Seitoku University, Tokyo, Japan

**Keywords:** adolescence, early life stress, elevated plus-maze, fear conditioning, forced swim, open-field, positive emotion, ultrasonic vocalizations

## Abstract

Social interactions are essential for neuronal development and emotional behavior. In particular, play behavior during adolescence facilitates neural and social development, whereas social isolation is detrimental and increases stress vulnerability in rodents. Furthermore, stress exposure during the juvenile and adolescent periods increases the risk of post-traumatic stress disorder in adulthood. Adolescent male rats emit 50-kHz ultrasonic vocalizations that reflect positive affect induced by rough-and-tumble play or tickling. We previously demonstrated that tickling, which mimics play behavior, induces positive affect and modulates fear-related behaviors as well as sympathoadrenal stress responses. Building on these findings, the present study investigated whether positive emotional experiences induced by this tickling procedure during adolescence can mitigate with long-term alterations in stress vulnerability following early-life stress exposure at weaning. Male Fischer rats were reared under three conditions from postnatal day 21: group-housed (three per cage), isolated (one per cage), or tickled (isolated and receiving 5 min of daily tickling stimulation). In adulthood, animals were subjected to a battery of behavioral tests, including the open-field, elevated plus-maze, forced swim, and fear conditioning tests. In rats that were socially isolated after weaning, repeated tickling incorporating handling during adolescence was associated with fewer behavioral alterations induced by juvenile foot-shock exposure and social isolation, particularly in the forced swim and fear conditioning tests. These findings suggest an association between positive experiences during adolescence and more favorable emotional outcomes following early-life stress.

## 1 Introduction

Adolescence is a critical period for the healthy development of body and mind. Chronic and/or extreme early-life stress disrupts brain maturation at structural and functional levels, increasing stress vulnerability to depression and post-traumatic stress disorder (PTSD) (Eiland and Romeo, 2013; Xie et al., 2023). In parallel with human studies, rodent findings have indicated that early-life stress experience impairs neural development (Zhao et al., 2020) and aggravates anxiety- and depressive-like behaviors (Cotella et al., 2019; Zhao et al., 2020). For example, rats repeatedly exposed to severe foot-shocks during adolescence exhibited heightened anxiety-related phenotypes, along with reduced dendritic length and number (Zhao et al., 2020). Conversely, positive experiences, such as physical exercise, reminiscing about positive memories, and social support, mitigate depressive or PTSD-like features induced by early-life stress in both humans (Askelund et al., 2019; Hegberg et al., 2019; Metts et al., 2023) and rodents (Ramirez et al., 2015; Burke et al., 2017; Zolfaghari et al., 2021). Positive experiences during adolescence can prevent stress vulnerability and enhance well-being in individuals exposed to early traumatic stress.

In male rats, rough-and-tumble play is frequently observed during the adolescent period [postnatal day (PND) 28–56] (Panksepp et al., 1984), which influences the establishment of following emotional and cognitive capacities, and normal sociability (Vanderschuren and Trezza, 2014). Indeed, depriving of social play leads to social (Hol et al., 1999) and cognitive impairments (Quan et al., 2010), and increases depressive-like (Leussis and Andersen, 2008; Cuesta et al., 2020; Dimonte et al., 2023) and anxiety-related phenotypes (Da Silva et al., 1996). Experimenters can artificially mimic social play by providing tickling stimulation, replicating dorsal contact and pinning behaviors (Burgdorf and Panksepp, 2001; Hori et al., 2013b,^2014^). Tickling stimulation elicits 50-kHz ultrasonic vocalizations (USVs) that reflect positive emotional states (Simola and Brudzynski, 2018), similar to social interaction during the rough-and-tumble play (Knutson et al., 1998). On the other hand, previous studies (Ishiyama and Brecht, 2016) have shown that anxious rats do not emit 50-kHz ultrasonic vocalizations (USVs) during handling alone. In addition, rats emit 50-kHz USVs when approaching the experimenter’s hand during tickling sessions, even in the absence of physical contact. These findings indicate that the behavioral and emotional responses elicited by tickling cannot be explained solely by handling. Therefore, the present study was not designed to examine the enrichment-like effects of handling per se, but rather to investigate the effects of juvenile play-like experiences that induce positive affective states in rats. Such positive experience during adolescence ameliorates heightened fear responses (Hori et al., 2013b) and cognitive impairments in isolated male rats (Hori et al., 2014). Thus, tickling stimulation is an effective treatment to prevent emotional and cognitive abnormalities in socially deprived individuals.

In our previous study, adolescent tickling ameliorated the delayed acquisition observed in the Morris water maze in socially isolated rats during adulthood, but not during late adolescence, whereas no clear group differences were detected in the probe test (Hori et al., 2014). These findings suggested that spatial memory retention was relatively preserved and raised the possibility that the beneficial effects of tickling may not be limited to cognitive function but may also involve long-term changes in emotional responsiveness, such as anxiety or stress reactivity.

Therefore, the present study was designed to investigate whether positive emotional experiences induced by tickling during adolescence ameliorate the detrimental effects of juvenile stress exposure on emotional behaviors in adulthood. To address this question, we employed a battery of behavioral tests assessing anxiety-like, depression-like, and fear-related behaviors. Tickling is a procedure that mimics social play behavior and is more effective in isolated rats than group-housed ones (Panksepp and Burgdorf, 2000). Additionally, early-life stress is sensitive to housing conditions (Vargas et al., 2016). Therefore, we established three housing conditions: group-housed, isolated, and isolated with tickling treatment. Using these experimental groups, we examined the effects of juvenile foot-shock stress on adult emotional behaviors and determined whether positive emotional experiences during adolescence could ameliorate the long-term adverse consequences of early-life stress.

## 2 Materials and methods

### 2.1 Animals

Male Fischer F344/NSlc rats were obtained at postnatal day (PND) 21 from Japan SLC Inc. (Shizuoka, Japan). The animals were housed either individually or in groups of three within standard polycarbonate cages (single: W170 mm × L330 mm × H200 mm; group: W230 mm × L380 mm × H200 mm) with wood chip bedding. The animals were maintained in a temperature- and humidity-controlled environment with a 12-h light/dark cycle (lights on at 08:00) and ad libitum access to food and water throughout the experiments. The cages of the isolated rats were shielded with paper screens to eliminate neighborhood’s sights; however, olfactory and auditory stimuli from other animals were not entirely eliminated. All experiments were approved by the University of Tsukuba Committee on Animal Research (Approval No. 21-414).

### 2.2 Experimental design

**FIGURE 1:**
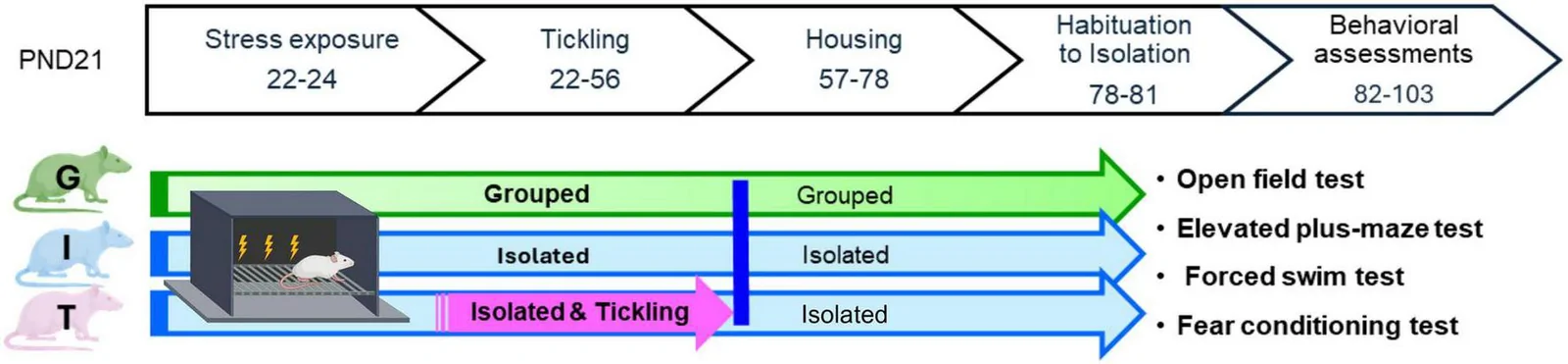
Experimental schedule. The rats used in the experiment were assigned to one of three conditions – group housing, individual housing, or individual housing with tickling experience – on postnatal day 21 (PND 21). Over the following 3 days, half of the animals in each condition were subjected to electric foot shocks. For the individual housing with tickling experience groups, tickling stimulation was administered until PND 56. The 3 days prior to behavioral experiments, the subject rats were habituated to isolation procedure. Open-field, elevated-plus maze, forced swim, and fear conditioning tests were conducted when the rats were between 82 and 103 days of age.

In the current study, we aimed to evaluate the effects of tickling treatment on emotional and stress-related behaviors in rats subjected to multiple aversive events: juvenile foot-shock exposure and adolescent social isolation. Accordingly, the rats were divided into six experimental groups: group-housed control (GC, *n* = 9), group-housed and shock-experienced (GS, *n* = 9), isolated control (IC, *n* = 9), isolated and shock-experienced (IS, *n* = 9), isolated and tickling-experienced (IT, *n* = 9), isolated and shock- and tickling-experienced (IST, *n* = 9). The shock-experienced groups were subjected to the 3-day foot-shock exposure described below (Figure 1). The rats in the IT and IST groups received tickling treatment described below until they reached 8-weeks-old (Figure 1). All rats were briefly removed from their home cages once weekly for routine husbandry procedures, including bedding replacement and body weight measurement, from juvenile foot-shock exposure until the end of the adult behavioral experiments. During the adult behavioral experiments, all rats were also transferred from their home cages to the respective testing apparatuses as required for each behavioral test. Apart from these routine husbandry procedures and transfers associated with behavioral testing, rats in the group-housed (GC, GS) and isolated (IC, IS) groups did not receive any additional physical contact or tactile stimulation. In contrast, rats assigned to the tickling groups (IT and IST) received repeated play-mimicking tickling sessions during adolescence. Therefore, the repeated tickling procedure inherently included both the play-mimicking tactile stimulation itself and the physical contact associated with transferring the rats from their home cages to the tickling chamber.

### 2.3 Juvenile foot-shock exposure

At PND 22–24, the rats assigned to the GS, IS, and IST were exposed to foot-shocks. On each day, the rats were individually placed in a quadrangular fear conditioning chamber as described previously (Hori et al., 2013b,^2014^) for a total of 730 s. Following the baseline in the first 5 min, five foot-shocks (0.5 mA intensity, 2-s duration) were presented to the rats with 30-s inter-shock intervals. Five min after the final shock presentation, the rats were removed from the chamber. The rats in the control groups (GC, IC, and IT) were placed in the fear conditioning chamber for the same duration as the other groups.

During the juvenile foot-shock exposure period, immediately following each foot-shock session, rats in the IST and IT groups received 5 min of tickling stimulation, and approach latency time (ALT) was measured before and after the stimulation in accordance with our previous reports. The isolated groups (IC, IS) were placed in the tickling stimulation chamber for the same duration, and their ALT was measured. The group-housed rats (GC, GS) were not placed in the tickling chamber and were returned directly to their home cages without receiving tickling stimulation.

### 2.4 Tickling treatment

Tickling stimulation was conducted by a single experimenter using a procedure described previously (Hori et al., 2013a,b,^2014^). For each session, rats were placed individually in a treatment box (270 × 440 × 187 mm polycarbonate cage) lined with a soft black cloth. Each rat received two 2-min tickling sessions separated by a 1-min interval. Each session consisted of four alternating cycles of 15 s of stimulation followed by 15 s without stimulation. Tickling was administered once daily for 5 consecutive days per week (Monday–Friday) over a 4-week period (PND 22 to PND 56). To evaluate the effects of tickling incorporating handling stimulation, ultrasonic vocalizations (USVs) emitted during one weekly tickling session were recorded and analyzed. In addition, ALT was measured as an index of tickling’s positive affective value. ALT was defined as the latency to approach the experimenter’s hand at the end of each tickling session, as described in our previous studies. During the juvenile foot-shock exposure period only, ALT was additionally measured before each tickling session, and both pre- and post-stimulation values were recorded. The maximum latency recorded was 30 s.

### 2.5 Ultrasonic vocalization (USV) recording and analysis

The USVs and their frequency characteristics were analyzed following the methodologies detailed in our previous reports (Hori et al., 2013a,^2014^; Shimoju et al., 2020) and Avisoft SASLab Pro (Avisoft Bioacoustics, Berlin, Germany). Each call was visually and acoustically identified by a trained observer on the spectrogram, using a minimum call duration of 10 ms and a maximum interruption of 10 ms between consecutive sound elements. The 50-kHz USVs were categorized according to a modified 10-subtype scheme (Composite, Upward ramp, Downward ramp, Flat, Short, Multi step, Step up, Step down, Trill, Inverted U) described by Wright et al. (2010).

### 2.6 Behavioral experiments

The subject rats engaged in a series of behavioral tests, including open-field (PND 82), elevated plus-maze (PND 83), forced swim (PND 85–86), and auditory fear conditioning and extinction (PND 96–103) (Figure 1). Prior to the beginning of the behavioral tests, all groups of rats were habituated to separation procedure for 3 days, taking into account that short-term isolation as an environmental change may influence behavioral alterations and stress responsiveness in group-housed rats (Komorowska and Pisula, 2003; Burman et al., 2014). During habituation, each rat was placed individually in the tickling chamber for 5 min per day and then returned to its home cage.

#### 2.6.1 Open-field test

The Behavioral test procedure and analysis are based on a previous report (Mochizuki-Kawai et al., 2022). Following the 3-day habituation to the separation procedure, the rats were introduced into a polyvinyl open-field arena (90 × 90 × 45 cm) at a light intensity of 59.5 lux and allowed to freely explore the arena for 5 min. Animal tracking was performed using the center point of the rat as the tracking reference. Distance traveled, time spent in the center area, rearing (unsupported rearing), and self-grooming behaviors were observed using the program Image OF (O’Hara & Co. Ltd, Tokyo, Japan), a modified software based on the public domain NIH Image program (developed at the US National Institutes of Health).

#### 2.6.2 Elevated plus-maze test

The rats were placed on the center platform of an elevated plus-maze apparatus (Toyoshima et al., 2018; Mochizuki-Kawai et al., 2022) and allowed to freely explore the maze for 5 min under a room light. Their behaviors were recorded by videotape for subsequent scoring. Animal tracking was performed using the center point of the rat as the tracking reference with DeepLabCut (Version 2.3.9). Total distance traveled in the entire area, time spent, and distance traveled in the open arms were measured based on the tracked center point. The network was trained using approximately 200 frames from all videos for 100,000 iterations. The data were imported into Python (Version 3.8.19) and processed using custom scripts.

#### 2.6.3 Forced swim test

The rats were individually subjected to 15 min pre-exposure in transparent cylinders (24-cm diameter and 45-cm height) filled with 35 cm of water at a temperature of 23 ± 1 °C. The animals could neither escape the water-filled cylinder nor support themselves by touching the bottom of the cylinder. The following day, the rats were subjected to a 5-min swim test in the same cylinder. Their behaviors were recorded on videotape, and the time spent immobile for each rat was scored by an experienced observer who was blinded to the treatment conditions using Any-maze video tracking software (Version 6.16, Stoelting Co., IL, USA). Immobility was defined as motionless, horizontal floating on the surface of the water, without movement of both the front and hind legs for over 2 s.

#### 2.6.4 Auditory fear conditioning and extinction

Auditory fear conditioning was conducted according to our previous reports (Hori et al., 2013b,^2014^) with some modifications. Briefly, each rat was exposed to five foot-shocks (0.5 mA intensity, 2-s duration) paired with tone cues (10 KHz, 65 dB, 10-s duration) after a 120-s baseline phase in the same conditioning chamber used for the juvenile foot-shock exposure. There was a 60-s interval between an offset of each shock presentation and onset of the next tone presentation. Sixty seconds after the final tone-shock pairing, the rat was removed from the chamber. Beginning the next day, the fear extinction procedure was conducted for 7 days in the triangular prism-shaped box (33 × 33 × 32 cm). The rats were placed in a triangle chamber for 120 s, and then exposed to two tone cues (10 KHz, 65 dB, 10-s duration) with a 120-s inter-tone interval. The percentage of time spent freezing behavior was calculated for each 30-s observation period using Image J FZ1 (O’Hara & Co., Ltd., Tokyo, Japan), a modified software based on the public domain NIH Image program.

### 2.7 Statistical analysis

All data are expressed as the means ± SEM of individual values for the rats. USV subtypes during tickling were analyzed using a multivariate analysis of variance (MANOVA) with juvenile foot-shock experience (shocked vs. non-shocked) as the between-subject factor and subtype counts as dependent variables. To characterize temporal changes during repeated tickling treatment, USV counts, duration, and each USV subtype (call counts and duration) were analyzed longitudinally using two-way mixed-design ANOVAs to evaluate main effects and interactions, with foot-shock condition as the between-subject factor and Week (Week 1–5) as the within-subject factor. Mauchly’s test was used to assess the assumption of sphericity, and Greenhouse–Geisser corrections were applied when sphericity was violated. ALT was analyzed using a three-way mixed ANOVA to evaluate main effects and interactions, with time (before vs. after tickling) as the within-subject factor and group (isolated vs. tickled) and juvenile foot-shock experience (shock vs. non-shocked) as between-subject factors. To evaluate the stability of ALT following juvenile foot-shock exposure, ALT measured at the end of tickling over the subsequent 3 days was analyzed using a repeated-measures ANOVA to evaluate main effects and interactions, with Day as the within-subject factor and group and juvenile foot-shock experience as between-subject factors. In the open-field were analyzed using two-way ANOVA to evaluate the main effects of and interactions between group (group-housed, isolated, or tickled) and juvenile foot-shock experience (shocked vs. non-shocked) as the between-subjects factors. One rat in the shocked and tickling-experienced group was identified as an outlier by the Grubb’s test and was therefore excluded from the analysis for time spent in the central area using R (4.2.2). In the elevated plus-maze, and forced swim tests, data were analyzed using two-way ANOVA to evaluate the main effects of and interactions between group (group-housed, isolated, or tickled) and juvenile foot-shock experience (shocked vs. non-shocked) as the between-subjects factors. Weight changes and freezing responses in the extinction phases were analyzed using a two-way repeated-measures mixed-design ANOVA to evaluate the main effects and their interactions, with group (group-housed, isolated, or tickled) and juvenile foot-shock experience (shocked or non-shocked) as the between-subjects factors and phase (Week 3–14, Day 1–7) as the within-subjects factor. When appropriate, *post hoc* comparisons were made with Bonferroni’s correction. Values of *P* < 0.050 were considered statistically significant (IBM SPSS Statistics Version 25.0, International Business Machines Co., New York, USA).

## 3 Results

### 3.1 Body weight

Body weight changes from baseline are shown in Supplementary Figure 1. A significant main effect of Week was observed (F_2.18_, _104.64_) = 9408.98, *p* < 0.001), indicating a progressive increase in body weight over time. There was also a significant Week × group interaction (F_4.36, 104.64_ = 3.02, *p* = 0.018), demonstrating that weight gain trajectories differed across housing conditions. In contrast, no significant Week × shock experience interaction was found (*p* = 0.660), and the three-way interaction was not significant (*p* = 0.104). For between-subject effects, a significant main effect of group was detected (F_2, 48_ = 3.42, *p* = 0.041), whereas no significant effect of shock experience (*p* = 0.352) was observed. The shock experience × group interaction showed a trend toward significance (*p* = 0.094).

*Post hoc* comparisons revealed that group-housed rats showed significantly higher body weight than tickled rats (*p* = 0.040). Further analyses of the Week × group interaction indicated that group-housed rats exhibited significantly greater body weight compared to tickled rats at several time points, particularly during mid-to-late developmental stages (e.g., Weeks 6–8 and Week 12; all *p* < 0.050), whereas no consistent differences were observed between group-housed and isolated rats.

### 3.2 Approach latency time

**FIGURE 2:**
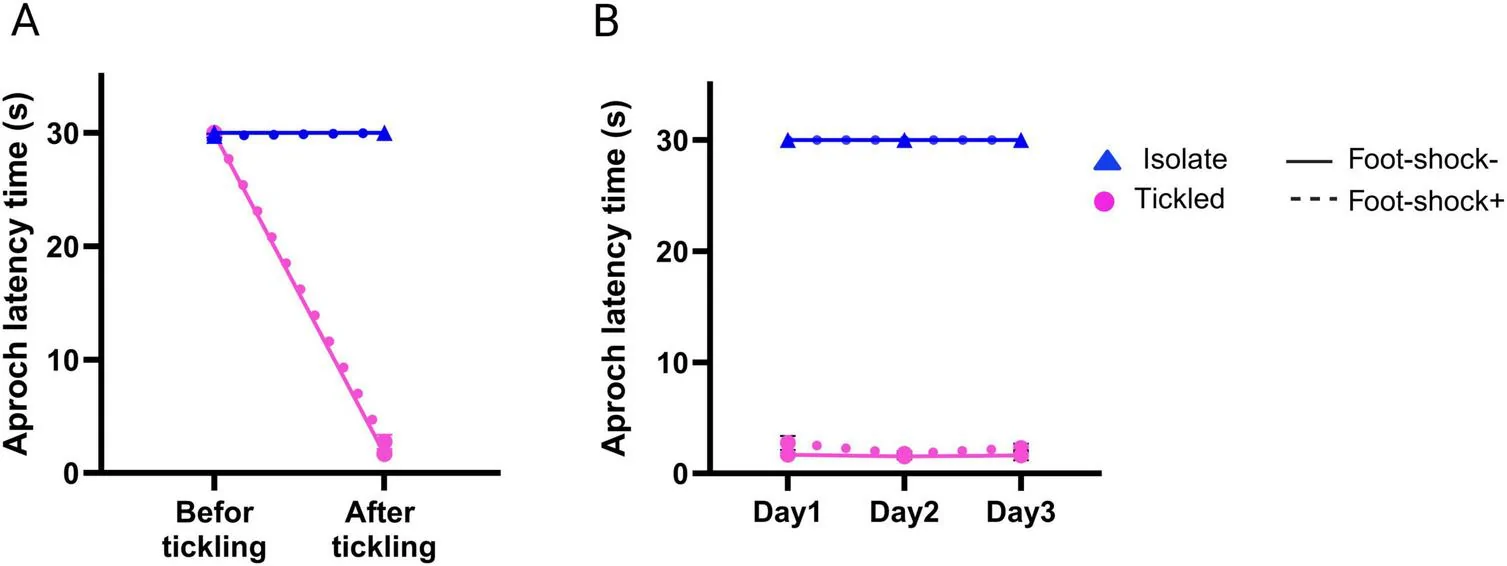
Approach latency. **(A)** Approach latency time before and after tickling on the first day of juvenile foot-shock exposure. **(B)** ALT after subsequent juvenile foot-shock sessions over the following three days. Data are shown as mean ± SEM. Data are shown as mean ± SEM.

Approach latency time (ALT) on the first day following juvenile foot-shock exposure is shown in Figure 2A. A three-way mixed ANOVA revealed significant main effects of time (*F*_1, 32_ = 6468.09, *p* < 0.001) and group (*p* < 0.001), whereas the main effect of shock experience was not significant (*p* = 0.193). Significant group × shock experience (*p* = 0.046) and time × group (*F*_1, 32_ = 6588.40, *p* < 0.001) interactions were observed, whereas the time × shock experience (*p* = 0.086) and time × group × shock experience (*p* = 0.311) interactions were not significant. The *post hoc* comparisons showed that ALT was significantly shortened after tickling (*p* < 0.001), whereas no change was observed in the isolation group (*p* = 0.602). No difference in ALT was detected between the groups before the intervention (*p* = 0.453); however, ALT was significantly shorter in the tickling-experienced group than in the isolated group after the intervention (*p* < 0.001). Furthermore, juvenile foot-shock significantly increased ALT in the Tickling-experienced group (*p* = 0.022), whereas no effect was observed in the isolated group (*p* = 0.600). Additionally, ALT at the end of tickling over the 3 days following juvenile foot-shock exposure is shown in Figure 2B. The tickling-experienced group exhibited significantly shorter ALT than the isolated group throughout the experiment. Accordingly, a significant main effect of group was observed (*F*_1, 32_ = 17589.78, *p* < 0.001). In contrast, there was no significant main effect of Day (*F*_2_, _64_ = 2.01, *p* = 0.142) or shock exposure (*F*_1, 32_ = 2.39, *p* = 0.132). Furthermore, neither the Day × group interaction (*F*_2_, _64_ = 2.01, *p* = 0.142), the Day × shock exposure interaction (*F*_2_, _64_ = 1.05, *p* = 0.358), nor the Day × group × shock exposure interaction (*F*_2_, _64_ = 1.05, *p* = 0.358) was significant.

### 3.3 USVs during tickling stimulation

**FIGURE 3:**
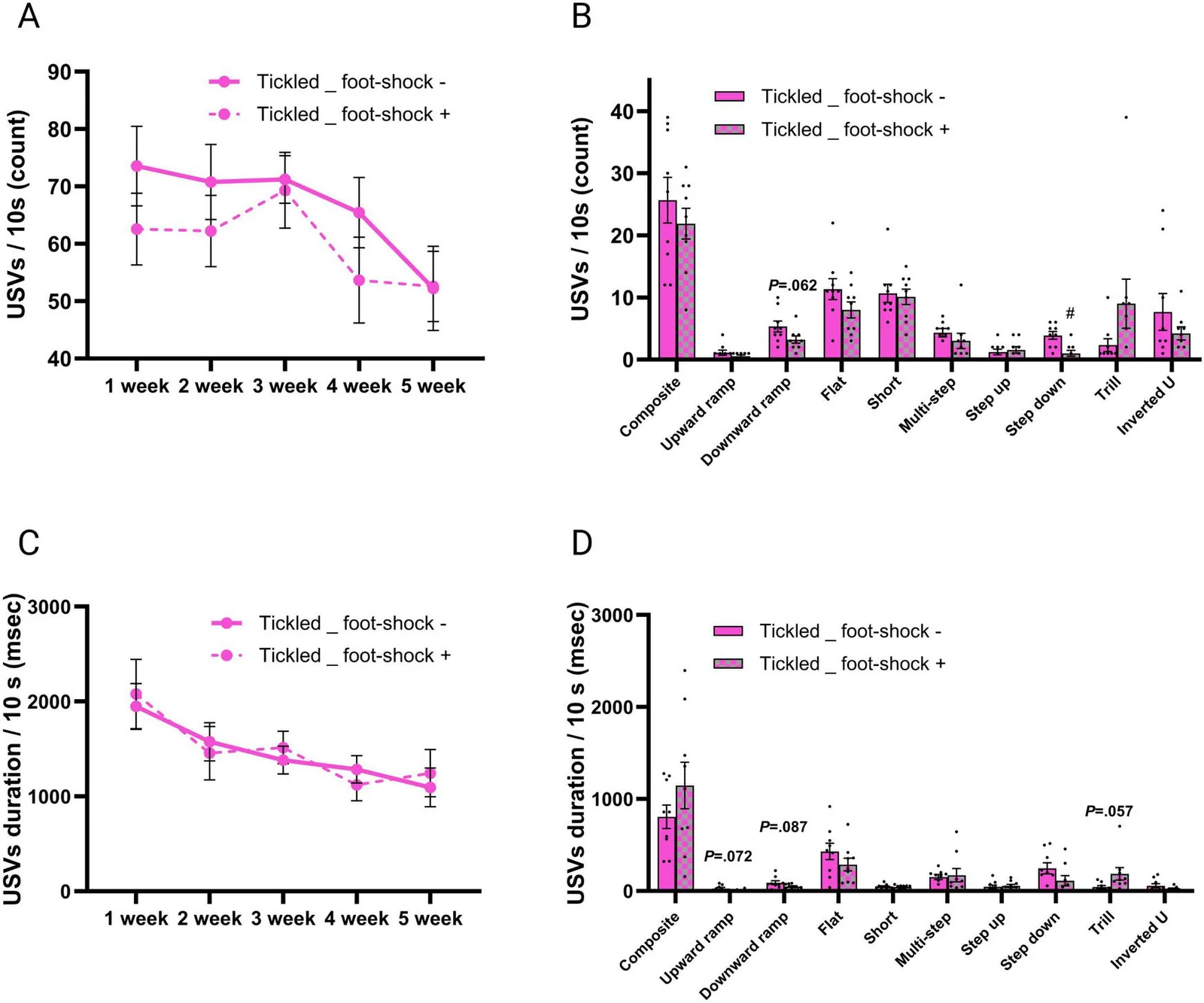
USVs during tickling stimulation. **(A)** Number of 50 kHz ultrasonic vocalizations (USV) over 5 weeks. **(B)** The number of calls of each USV subtype on the first day of juvenile foot-shock exposure. **(C)** Changes in ultrasonic vocalization durations over the 5-week period. **(D)** Classification of duration of USV subtypes on the first day of juvenile foot-shock exposure: duration. Data are shown as mean ± SEM. ^#^*p* < 0.05 indicate differences between foot-shock conditions within the same group.

Changes in ultrasonic vocalizations (USVs) over the 5-week period are shown in Figure 3A. For USV counts, a significant main effect of Week was found (Greenhouse–Geisser corrected: *F*_3.09,__49.43_ = 4.47, *p* = 0.007), reflecting a decline across weeks. There was no significant effect of shock experience (*p* = 0.339) or interaction between Week and shock experience (*p* = 0.606). Similarly, for USV duration, a repeated-measures ANOVA revealed a significant main effect of Week (Greenhouse–Geisser corrected: *F*_2.28, 36.4_0 = 6.99, *p* = 0.002), indicating a gradual decrease over time. No significant main effect of shock experience (*p* = 0.909) or their interaction (*p* = 0.725) was observed (Figure 3C).

The classification of USV subtypes on the first day of juvenile foot-shock is shown in Figure 3B. Juvenile foot-shock experience significantly affected USV subtype composition during tickling stimulation (Wilks’ Λ = 0.160, F_10, 7_ = 3.68, *p* = 0.049, η^2^ = 0.840). Shock experience tickled rats emitted fewer Step-down calls than non- shock experience rats (F_1, 16_ = 14.01, *p* = 0.002, η^2^ = 0.467), and a similar decreasing tendency was observed for Downward ramp calls (F_1, 16_ = 4.03, *p* = 0.062, η^2^ = 0.201), while no differences were found for other subtypes. However, this significant difference in call counts was no longer observed at Week 5 (Wilks’ Λ = 0.541, F_10, 7_ = 0.593, *p* = 0.781). In contrast, no significant effect of shock experience was observed on the duration of USV subtypes (Wilks’ Λ = 0.325, F_10, 7_ = 1.45, *p* = 0.319). No statistically significant differences were detected in individual subtypes; however, several showed moderate to large effect sizes, including Trill (η^2^ = 0.208, *p* = 0.057), Upward ramp (η^2^ = 0.188, *p* = 0.072), and Downward ramp (η^2^ = 0.172, *p* = 0.087) (Figure 3D).

To further characterize the temporal profile of USV subtype changes during repeated tickling stimulation, each subtype was analyzed longitudinally across the 5-week treatment period (Supplementary Table 1). Significant Week × shock experience interactions were detected for the Upward ramp subtype in both call counts (*F*_4, 64_ = 3.05, *p* = 0.023) and call duration (*F*_4, 64_ = 3.22, *p* = 0.018), as well as for Step-down call counts (Greenhouse–Geisser corrected: (*F*_2.36, 37.73_ = 3.78, *p* = 0.026). In contrast, Step-down call duration (Greenhouse–Geisser corrected: *p* = 0.085) and Trill call duration (Greenhouse–Geisser corrected: *p* = 0.061) showed trends toward a Week × shock experience interaction. *Post hoc* comparisons indicated that these interactions reflected different temporal changes between the groups during repeated tickling stimulation. However, the between-group differences gradually diminished over the course of the 5-week treatment, and no significant differences between the juvenile foot-shock and non-foot-shock groups remained by the end of the tickling period. The temporal changes in the Upward ramp, Step-down, and Trill call durations are illustrated in Supplementary Figure 2A–C, respectively. Apart from these subtypes, several USV subtypes, including Composite, Flat, Short, Multi-step, and Trill (call counts), showed significant changes across weeks regardless of juvenile foot-shock experience, whereas others remained stable throughout the experiment (Supplementary Table 1), suggesting that some USV subtypes change over weeks with development and/or repeated tickling, whereas others remain relatively stable.

### 3.4 Open-field test

**FIGURE 4:**
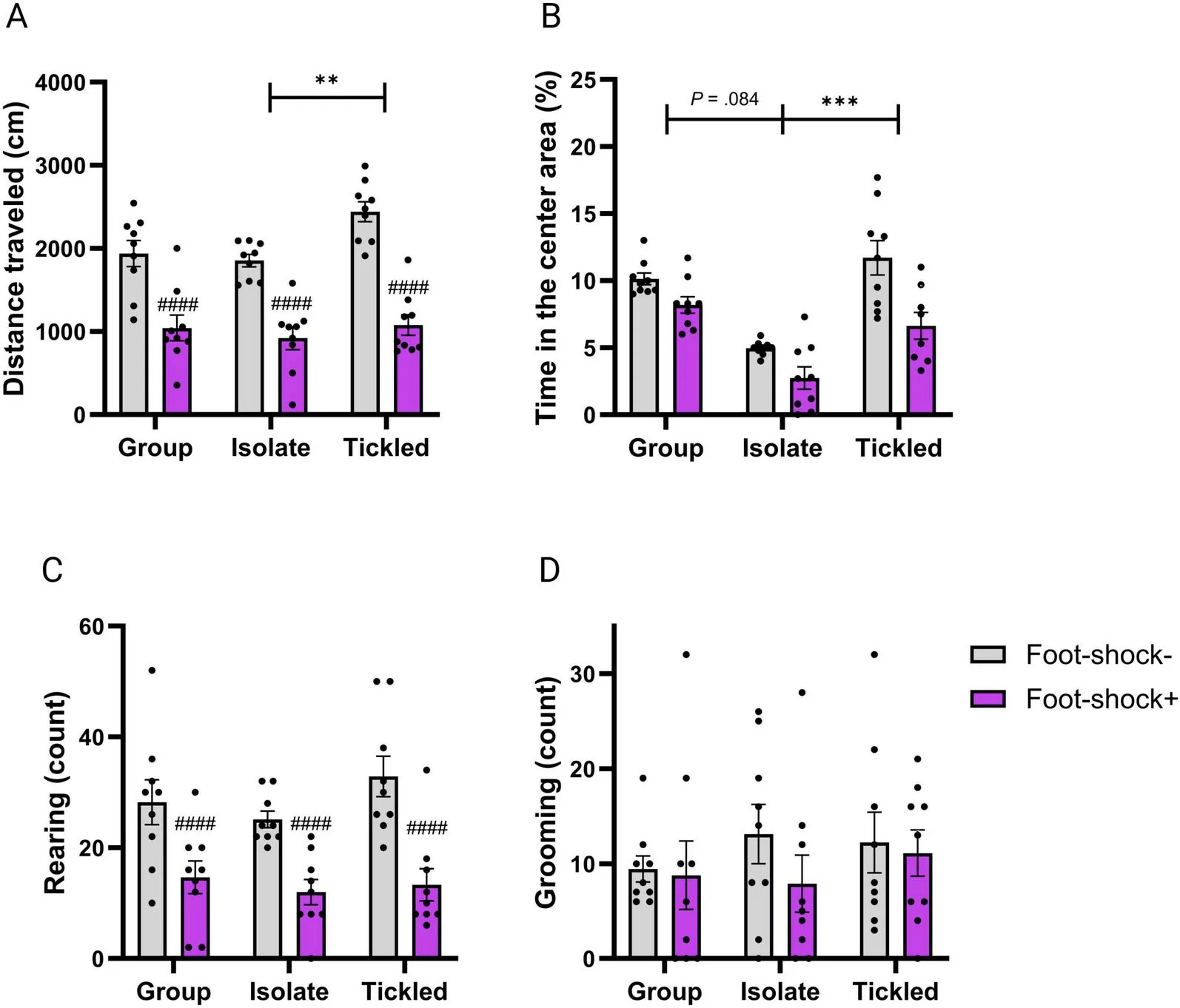
Open-field test. **(A)** Total distance traveled in the open-field apparatus. **(B)** Percentage of time spent in the center area. **(C)** Number of rearing behaviors. **(D)** Number of self-grooming. Data are shown as mean ± SEM. **p < 0.01, ***p < 0.001 indicate differences between groups. ^ ####^*p* < 0.0001 indicate differences between foot-shock conditions within the same group.

The distance traveled in the open-field arena is shown in Figure 4A. The rats in the shock-experienced groups exhibited less distance traveled than those in the non-shocked groups. Two-way ANOVA revealed significant main effects of group (*F*_2,48_ = 4.337, *p* = 0.019) and shock experience (*F*_1,48_ = 99.734, *p* < 0.001). The *post hoc* comparisons revealed that the distance traveled in the isolated plus tickling-experienced groups was significantly longer than that in the isolated groups (*p* = 0.002). The time spent in the center area of the open-field arena is shown in Figure 4B. Two-way ANOVA revealed significant main effects of group (*F*_2,47_ = 5.857, *p* = 0.005) and shock experience (*F*_1,47_ = 21.835, *p* < 0.001). The *post hoc* comparisons indicated that isolated rats spent less time in the center than both group-housed and tickling-experienced ones (isolated vs. group-housed: *p* = 0.084; isolated vs. tickling-experienced: *p* = 0.008). The number of rearing behaviors is shown in Figure 4C. The rats in the shock-experienced groups exhibited fewer rearing behaviors than those in the non-shocked groups. Two-way ANOVA revealed a significant main effect of shock experience (*F*_1,48_ = 39.429, *p* < 0.001). Figure 4D shows the number of self-grooming behaviors. Two-way ANOVA did not reveal significant main effects of group and shock experience, and their interaction.

### 3.5 Elevated plus-maze test

**FIGURE 5:**
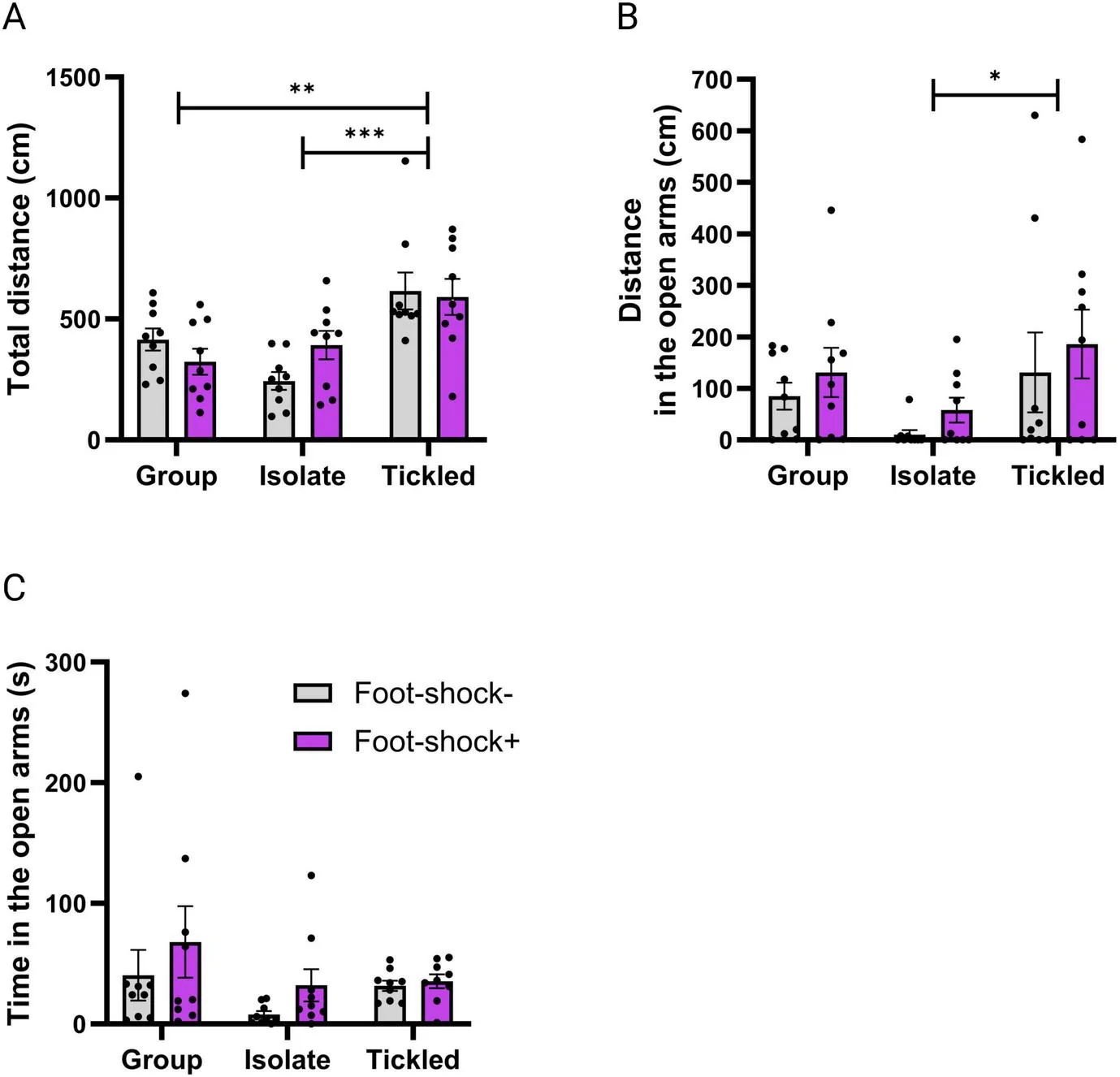
Elevated plus-maze test. **(A)** Total distance traveled in the entire area of the elevated plus-maze apparatus. **(B)** Distance traveled in the open arms. **(C)** Time spent in the open arms. Data are shown as mean ± SEM. *p < 0.05, **p < 0.01, ***p < 0.001 indicate differences between groups.

The total distance traveled in the entire apparatus is shown in Figure 5A. Regardless of early foot-shock experience, the rats in the tickling-experienced groups traveled a longer distance compared to those in the other conditions. Two-way ANOVA revealed a significant main effect of group (*F*_2,48_ = 13.20, *p* < 0.001). The *post hoc* comparisons revealed that the distance traveled in the tickling-experienced groups was significantly longer than that in the group-housed (*p* = 0.001) and isolated groups (*p* < 0.001). In distance traveled in the open arms (Figure 5B), two-way ANOVA also revealed a significant main effect of group (*F*_2,48_ = 3.32, *p* = 0.045). The *post hoc* comparisons revealed that the tickling-experienced rats traveled a longer distance than the isolated ones (*p* = 0.041). Figure 5C shows time spent in the open arms. Two-way ANOVA did not reveal significant main effects of group and shock experience, and their interaction.

### 3.6 Forced swim test

**FIGURE 6:**
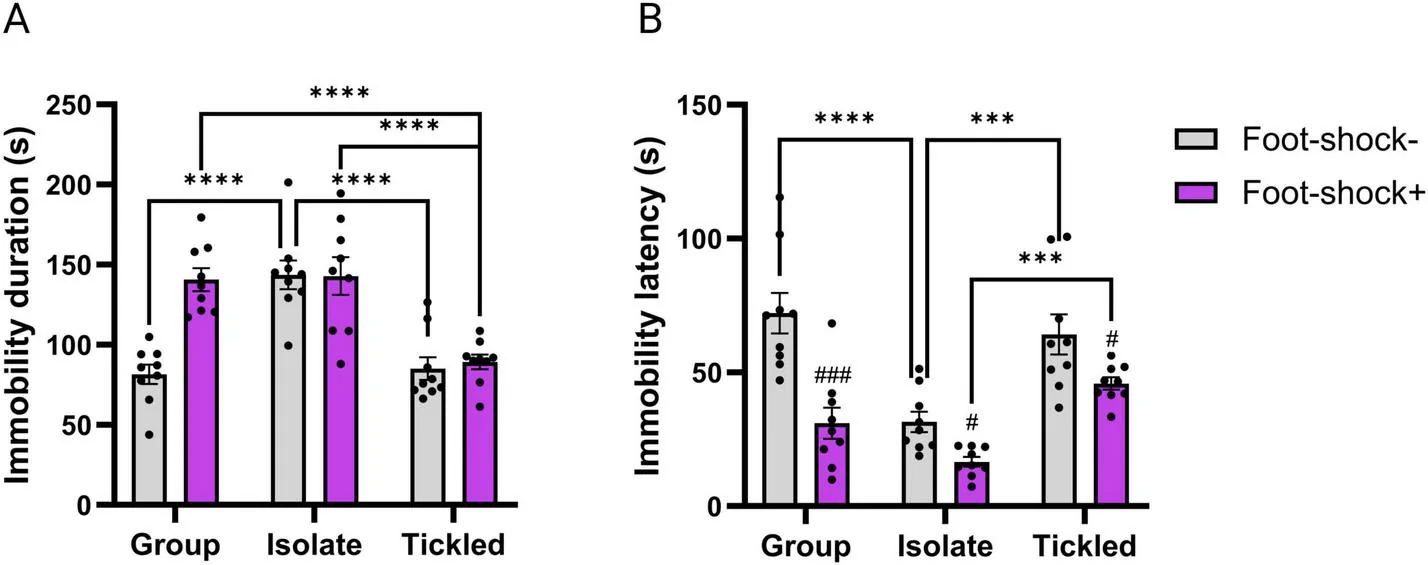
Forced swim test. **(A)** Duration of immobility. **(B)** Latency to first immobility event. Data are shown as mean ± SEM. ***p < 0.001, ****p < 0.0001 indicate differences between groups within the same foot shock condition. ^#^*p* < 0.05, ^###^*p* < 0.001 indicate differences between foot-shock conditions within the same group.

The duration of immobility time is shown in Figure 6A. Two-way ANOVA revealed significant main effects of group (*F*_2,48_ = 25.214, *p* < 0.001), shock experience (*F*_1,48_ = 10.339, *p* = 0.002), and their interaction (*F*_2,48_ = 8.781, *p* = 0.001). The *post hoc* comparisons revealed that early shock exposure in the group-housed rats increased the duration of immobility time (*p* < 0.001). Additionally, in the non-shocked condition, isolated rats were more immobile than those in the other two groups (isolated vs. group-housed: *p* = 0.000; isolated vs. tickling-experienced: *p* < 0.001) whereas tickling-experienced rats exhibited lower immobility time compared to the other two groups in the shocked condition (isolated vs. group-housed: *p* < 0.001; isolated vs. tickling-experienced: *p* < 0.001). Figure 6B shows the latency to the first immobility in each experimental condition. Two-way ANOVA revealed significant main effects of group (*F*_2,48_ = 19.877, *p* < 0.001) and shock experience (*F*_1,48_ = 32.955, *p* < 0.001), and their interaction (*F*_2,48_ = 3.469, *p* = 0.039). The *post hoc* comparisons revealed that, in the non-shocked condition, the immobile latency in the isolated group was shorter than both in group-housed and tickling-experienced groups (isolated vs. group-housed: *p* = 0.000; isolated vs. tickling-experienced: *p* < 0.001) whereas it was shorter only than that in the tickling-experienced group in the shocked condition (isolated vs. tickling-experienced: *p* = 0.001). Additionally, significant differences in the immobile latency were observed between shocked and non-shocked conditions in all groups (group-housed: *p* < 0.001, isolated: *p* = 0.046, tickling-experienced: *p* = 0.019).

### 3.7 Auditory fear conditioning and extinction

**FIGURE 7:**
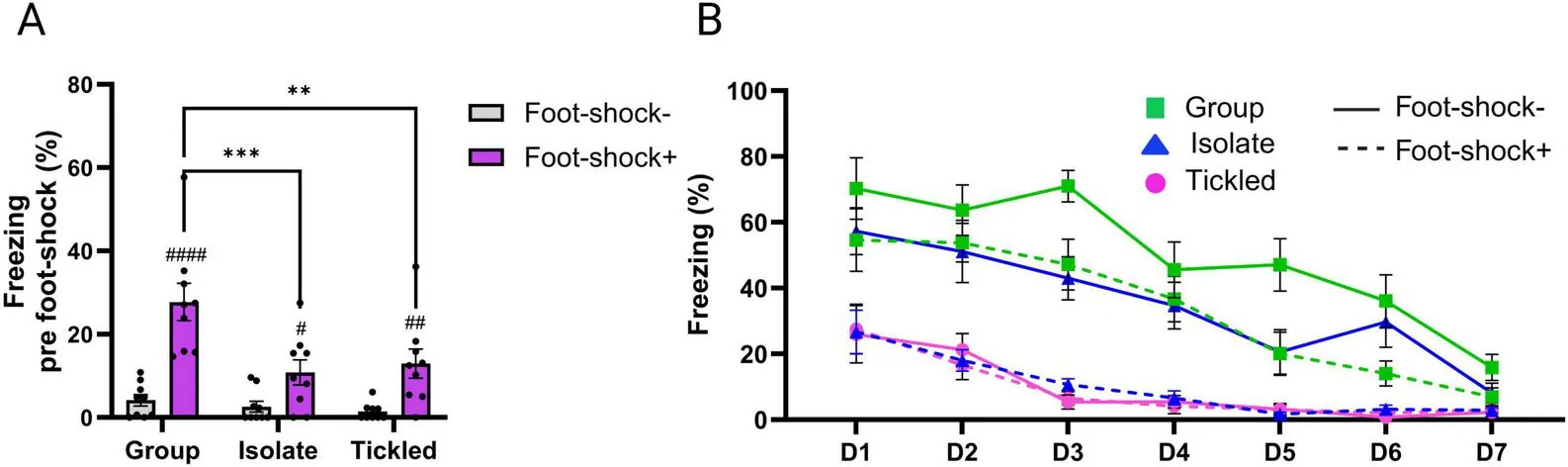
Auditory fear conditioning and extinction test. **(A)** Percentage of freezing response prior to shock exposure during the conditioning phase. **(B)** Percentage of freezing response during the extinction phases. Data are shown as mean ± SEM. **p < 0.01, ***p < 0.001 indicate differences between groups within the same foot shock condition. ^#^*p* < 0.05, ^##^*p* < 0.01, ^####^*p* < 0.0001 indicate differences between foot-shock conditions within the same group.

The percentage of freezing during pre-shock exposure in the conditioning phase is shown in Figure 7A. According to two-way ANOVA, there were significant main effects of group (*F*_2,48_ = 7.156, *p* = 0.002) and shock experience (*F*_1,48_ = 41.538, *p* < 0.001), and their interaction (*F*_2,48_ = 4.323, *p* = 0.019). The *post hoc* comparisons revealed that early shock exposure increased freezing behaviors in each group (group-housed: *p* < 0.001, isolated: *p* = 0.039, tickling-experienced: *p* = 0.005). Additionally, the group-housed rats exhibited higher freezing level compared to the isolated (*p* < 0.001) and tickling-experienced ones (*p* = 0.001) in the shocked condition. Figure 7B shows the percentage of freezing during extinction phases. According to three-way repeated measure ANOVA, there were significant main effects of group (*F*_2,48_ = 63.145, *p* < 0.001), shock experience (*F*_1,48_ = 33.086, *p* < 0.001), and Day (*F*_2,48_ = 135.178, *p* < 0.001) and interaction (*F*_2,48_ = 7.771, *p* = 0.001). In the group-housed rats, freezing responses were different between the non-shocked and shocked conditions on Day 3 (*p* = 0.002), Day 5 (*p* = 0.001), Day 6 (*p* = 0.007), and Day 7 (*p* = 0.009). Regarding the isolated groups, freezing levels between the shocked and non-shocked conditions were significantly different during the extinction (*p*’s < 0.05) except Day7 (*p* = 0.170). On the other hand, no significant differences between the non-shocked and shocked conditions in the tickling-treated groups were observed throughout the extinction (all *p*’s > 0.05).

## 4 Discussion

In the present study, we have examined whether early-life stress experience aggravates emotional behaviors, and those detrimental effects could be reversed by adolescent tickling experience. The present study demonstrates that, under conditions of post-weaning social isolation, positive emotional experiences during adolescence, such as those induced by a tickling procedure that mimics social play, were associated with reduced anxiety- and depressive-like behaviors in adulthood, particularly in rats exposed to juvenile foot-shock stress. These findings are similar to the beneficial effects observed when stress-related behavioral changes were mitigated by restoring social interaction following isolation (Whitten et al., 2025) and extend our previous studies (Hori et al., 2013b,^2014^) by showing that the long-term benefits of adolescent tickling are not limited to cognitive performance but are also associated with adult emotional behaviors. Together, these findings support the idea that positive emotional experiences during adolescence may influence long-term emotional outcomes following early-life stress. Our findings indicate that housing conditions, but not shock experience, modulate anxiety-like behaviors, while there were additive effects of juvenile shock experience and adolescent housing conditions on depressive-like and fear responses.

Juvenile shock exposure and adolescent housing conditions independently influence general activity levels. Specifically, shock experience decreases the distance traveled in the open-field arena, consistent with the previous finding of chronic restraint stress exposure during juvenility (Pinzón-Parra et al., 2019). Regarding housing conditions, rats with tickling experience exhibited greater locomotor activity as distance traveled in the open-field test compared to isolated, but not group-housed rats. Similarly, increased locomotion in the tickling-experienced group was also observed in the elevated plus-maze test. Consistent with previous studies (Lukkes et al., 2009; Mora-Gallegos and Fornaguera, 2019), comparable locomotor activity levels were found between group-housed and isolated animals. Our data suggest that adolescent tickling, involving handling, enhanced overall locomotor activities.

Anxiety-related phenotypes in the open-field are modulated by housing but are not affected by shock experience. In the open-field test, isolated rats spent less time in the center area compared to group-housed ones, corroborating previous findings (Arakawa, 2003; Baghaei Naeini et al., 2023). Furthermore, the current results show tickling-experienced rats spent more time in the center compared to isolated rats, indicating that tickling treatments ameliorate isolation-heightened anxiety level. In contrast to the results in the open-field, housing conditions did not influence anxiety-like behaviors in the elevated plus-maze. This discrepancy between the open-field and elevated plus-maze tests may be attributed to differences in the constructions of the apparatuses. Figueiredo Cerqueira et al. (2023) have mentioned that the elevated plus-maze apparatus enables animals to be more anxious than the open-field, since they are suspended and face cliffs when exploring the open arms. Additionally, in the present study, the open-field test was conducted in dim light, while the elevated plus-maze test was carried out in room light. The differences in the light intensity in test situations may also contribute to different impact of social isolation on anxiety-related features. Taken together, our results expand findings that isolation experience during adolescence partly increases anxiety level, and tickling, involving handling, can reverse these adverse effects. Rats subjected to tickling exhibited significantly shorter approach latency times (ALT) than isolated rats, indicating that tickling produced a positive reward effect, consistent with our previous findings. This effect was independent of juvenile foot-shock experience, as no main or interaction effects were observed on ALT. Although both the number and duration of 50-kHz USVs gradually decreased over the 5-week treatment period, this decline was independent of juvenile foot-shock experience and likely reflected developmental changes and/or habituation to repeated tickling stimulation. Longitudinal analyses showed that juvenile foot-shock influenced the temporal profiles of only a limited number of USV subtypes, whereas most subtypes changed similarly over time regardless of foot-shock experience. Importantly, these differences gradually diminished and were no longer evident by Week 5, indicating that juvenile foot-shock had only a transient effect on positive affect-related vocalizations and did not produce long-lasting alterations in 50-kHz USV expression.

General motivation and exploratory behaviors are modulated by shock experience independent of housing conditions. All groups of the shocked condition showed fewer rearing behaviors than those of the non-shocked condition in the open-field test. These tendencies correspond with previous finding that chronic variable stress exposure (Luo et al., 2014; Xu et al., 2015) as well as inescapable foot-shocks (Zhao et al., 2020) decrease rearing behaviors. On the other hand, isolation housing did not influence rearing behaviors. Indeed, the effects of housing conditions on rearing behaviors remain controversial. Some reported that social isolation increased rearing behaviors (Dalrymple-Alford and Benton, 1981; Varty et al., 2000), while others showed no effect (Ferdman et al., 2007) in male rats. Furthermore, the present study demonstrates that the number of rearing behaviors in the tickling-experienced rats is not different from the group-housed and isolated rats, implying that tickling incorporating handling cannot recover fewer rearing behaviors induced by juvenile shock exposure. Overall, juvenile shock experience impairs exploratory motivation, which cannot be reversed by later tickling treatments, similar to group housing.

In depressive-like behaviors in the forced swim test, the additive effects between housing conditions and juvenile shock experience were observed. Among the non-shocked groups, the isolated rats exhibited longer immobility duration compared to the group-housed and tickling-experienced ones, similar to previous findings that isolation housing during adolescence increases immobility duration (Brenes et al., 2020; Song et al., 2021). Additionally, immobility latency in the isolated rats was shorter than those in the other two conditions. These results imply that isolated rats are more susceptible to have a depressive-like trait that can be prevented by tickling treatment. Comparing between the non-shocked and shocked conditions in each housing condition, juvenile shock exposure increased immobility duration in the group-housed rats, like previous studies (Lyttle et al., 2015; Aikawa et al., 2020). On the other hand, tickling-experienced rats showed fewer immobility and relatively longer latency even if receiving foot-shocks during juvenility. Notably, under the shocked condition, the latency to immobility significantly differed between the isolated and tickling-experienced groups, suggesting that experience of tickling associated with handling during adolescence can modify the stress reactivity exacerbated by the combination of early-life foot-shock and isolation. These results highlight that juvenile foot-shock exposure enhances a depressive-like state independent of housing environment, whereas tickling incorporating handling effectively prevents depression in individuals that have experienced early-life stress.

Juvenile shock experience increases basal freezing responses in fear conditioning test in adulthood. Rats that experienced foot-shocks during juvenility, especially in the group-housed, expressed higher freezing behaviors prior to the foot-shock exposure in the conditioning phase. In fact, the present study conducted fear conditioning for the adult rats in the same spatial context as juvenile shock exposure. Thus, these contextual cues could remind them of their fears when they were exposed to the context again in adulthood. Additionally, among the shocked groups, the freezing level during the pre-shock phase in the group-housed rats was higher than those in the isolated ones. Previous studies have documented that isolation housing reduced conditioned freezing responses (Rudy et al., 1999; Okada et al., 2014, 2015; Tamura et al., 2024). To our knowledge, no studies have been reported that confirm whether isolation rearing modifies the maintenance of fear memories in juvenility or inhibits the retrieval of them in adulthood. Diminished freezing behaviors during the pre-shock presentation period in the shock-experienced isolated groups may attribute to the possibility that isolation housing weakens the ability to memorize or retrieve foot-shock experience or fear-related cues resulting in mitigating fear expression. Furthermore, freezing levels in the tickling-experienced group were lower than that in the group-housed, similar to the isolated group. This finding suggests that adolescent tickling, involving handling, did not alter the reduction in isolation-induced fear- or anxiety-related responses that would reflect potential alleviating properties of tickling to the memory impairment associated with juvenile foot-shock experience.

Housing conditions and shock experience modulate freezing responses during the fear extinction. The day after the fear conditioning (extinction day 1), the group-housed rats exhibited higher, whereas the tickling-experienced rats show lower, freezing levels independent of the presence of shock experience. In addition, freezing responses in the tickling-experienced groups consistently lower than group-housed ones throughout extinction. Our previous study has reported that conditioned freezing responses in the group-housed condition were higher than tickling-experienced group (Hori et al., 2014). Thus, the tendency of consistent higher freezing in the group-housed rats was robust. Indeed, we have previously showed no differences in pain sensitivity among housing conditions (Hori et al., 2014). Therefore, the differences in fear rather than in pain sensitivity would explain enhanced freezing behaviors in the group-housed rats. These results suggest that repeated tickling incorporating handling may increase tolerance to fear. Furthermore, juvenile shock experience decreases freezing responses during the extinction in the group-housed and isolated groups. To our knowledge, direct evidence that prior foot-shock exposure during the developmental stage modulates later fear conditioning has not been provided. Besides, the effects of stress experience on following fear conditioning performance remain undetermined. Yee et al. (2012) reported that juvenile variable stress exposure potentiated conditioned freezing behaviors in adulthood. On the other hand, traumatic stress experience in adolescence suppressed fear responses and serum corticosterone levels following fear conditioning procedure (Moore et al., 2014). Regarding the later description, Moore et al. (2014) has mentioned two opponent possibilities; one is that early-life stress impairs the abilities to acquire, retain, and/or express conditioned fear, whereas the other is that early-life stress promotes resilience to later-life stress challenges. Although which is proper for our case cannot be determined, we speculate juvenile foot-shocks suppress aversive learning and memory abilities rather than mitigate stress responses because stress-related phenotype, such as depressive-like immobility, in this study was aggravated by early-life shock stress. Additionally, inescapable foot-shock exposure during juvenility disrupts performance in the Morris water maze (Lu et al., 2017), a learning and memory test under a stressful situation, suggesting that juvenile shock exposure may impair learning and memory abilities related to aversive emotion. To verify this hypothesis, future studies examining whether juvenile shock experience modulates water maze performance in group-housed and isolated conditions is needed.

Beyond the behavioral findings, the present study provides a broader perspective on the potential role of positive emotional experiences during adolescence in shaping long-term emotional outcomes following early-life stress. Our findings are consistent with the concept that positive social experiences during critical developmental periods may influence emotional resilience later in life. Because adolescence represents a period of heightened neural plasticity, experiences and environments that promote positive affect and social interaction during this stage may have long-lasting influences on emotional well-being. Although the present findings were obtained in an animal model, they highlight the importance of positive social experiences during adolescence as a potential factor associated with healthy emotional development. These observations may provide experimental support for strategies that promote social play, positive social interactions, and environmental enrichment during development as approaches to enhance emotional well-being and resilience following early-life adversity.

Several neurobiological mechanisms may underlie the long-term behavioral outcomes associated with adolescent tickling observed in the present study. In our previous studies, we demonstrated that tickling induces frequency-modulated (FM) 50-kHz ultrasonic vocalizations, which are widely recognized as indicators of positive affective states, together with increased dopamine release in the nucleus accumbens (Hori et al., 2013a). We further showed that repeated tickling is associated with long-lasting changes in brain gene expression (Hori et al., 2009) and enhanced adult hippocampal neurogenesis in the dentate gyrus (Yamamuro et al., 2010), suggesting that repeated positive tactile experiences can be associated with persistent neuroplastic changes. Recent studies have further expanded these findings by demonstrating that play and tickling responses are represented in the lateral columns of the periaqueductal gray (Gloveli et al., 2023), that oxytocin signaling facilitates human touch-induced play behavior in rats (Hayashi et al., 2025), and that rats genetically predisposed to depression exhibit altered ultrasonic vocalization responses during tickling (Kai et al., 2025). Taken together, these findings suggest that experience-dependent neural plasticity may represent one possible neurobiological basis for the long-term emotional behavioral changes observed in the present study. However, these neurobiological mechanisms were not directly examined here. Future studies combining behavioral, molecular, and neurophysiological approaches will be necessary to clarify the mechanisms underlying these long-term effects.

The present study has observed the influences of juvenile shock experience and adolescent housing environment on emotional behaviors in male rats. We demonstrated that repeated tickling incorporating handling during adolescence ameliorated the anxiety- and depressive-like behavioral alterations induced by social isolation, as evidenced by increased center exploration in the open-field test and reduced immobility in the forced swim test. Given that the previous study has shown that juvenile social play contributes to normal emotional development (Vanderschuren and Trezza, 2014), tickling stimulation may mimic social play and promote emotional development in isolated individuals. However, because the present study did not examine the effects of tickling in group-housed rats, it remains unclear whether tickling primarily compensates for the lack of social interactions under isolation or whether it represents a distinct positive emotional experience. Future studies including tickled group-housed rats will be necessary to distinguish between these possibilities. Overall, positive emotional experiences induced by physical interactions, including tickling and handling, may protect animals from developing anxiety- and depressive-like emotional states. Physical interactions with rats, including tickling and handling, during adolescence may contribute to recovery from adverse effects of early-life stress and/or adolescent social isolation.

## 5 Limitations

Several limitations should be considered. First, the juvenile foot-shock paradigm represents an acute and specific stressor, limiting the generalizability of the findings to other forms of early-life stress. Second, the behavioral measures employed may not fully capture the multidimensional nature of emotional processing. Furthermore, only male rats were used, precluding conclusions regarding sex differences. In addition, the absence of a non-tickled handling control group limits our ability to determine whether the observed behavioral differences were specifically attributable to tickling-induced positive affect or to other factors associated with handling and experimenter interaction. Finally, the absence of neuroendocrine or neural measurements restricts mechanistic interpretation. Future studies incorporating appropriate handling controls and neurobiological analyses will be necessary to clarify the mechanisms underlying the observed behavioral outcomes.

## Data Availability

The original contributions presented in the study are included in the article/Supplementary material, further inquiries can be directed to the corresponding author.

## References

[B1] Aikawa K. Yoshida T. Ohmura Y. Lyttle K. Yoshioka M. Morimoto Y. (2020). Subanesthetic ketamine exerts antidepressant-like effects in adult rats exposed to juvenile stress. *Brain Res*. 1746:146980. 10.1016/j.brainres.2020.146980 32544501

[B2] Arakawa H. (2003). The effects of isolation rearing on open-field behavior in male rats depends on developmental stages. *Dev. Psychobiol.* 43 11–19. 10.1002/dev.10120 12794774

[B3] Askelund A. D. Schweizer S. Goodyer I. M. Van Harmelen A.-L. (2019). Positive memory specificity is associated with reduced vulnerability to depression. *Nat. Hum. Behav.* 3 265–273. 10.1038/s41562-018-0504-3 30953005

[B4] Baghaei Naeini F. Hassanpour S. Asghari A. (2023). Resveratrol exerts anxiolytic-like effects through anti-inflammatory and antioxidant activities in rats exposed to chronic social isolation. *Behav. Brain Res.* 438:114201. 10.1016/j.bbr.2022.114201 36334782

[B5] Brenes J. C. Fornaguera J. Sequeira-Cordero A. (2020). Environmental enrichment and physical exercise attenuate the depressive-like effects induced by social isolation stress in rats. *Front. Pharmacol.* 11:804. 10.3389/fphar.2020.00804 32547399 PMC7272682

[B6] Burgdorf J. Panksepp J. (2001). Tickling induces reward in adolescent rats. *Physiol. Behav.* 72 167–173. 10.1016/S0031-9384(00)00411-X 11239994

[B7] Burke A. R. McCormick C. M. Pellis S. M. Lukkes J. L. (2017). Impact of adolescent social experiences on behavior and neural circuits implicated in mental illnesses. *Neurosci. Biobehav. Rev.* 76 280–300. 10.1016/j.neubiorev.2017.01.018 28111268

[B8] Burman O. Buccarello L. Redaelli V. Cervo L. (2014). The effect of two different individually ventilated cage systems on anxiety-related behaviour and welfare in two strains of laboratory mouse. *Physiol. Behav.* 124 92–99. 10.1016/j.physbeh.2013.10.019 24184492

[B9] Cotella E. M. Gómez A. S. Lemen P. Chen C. Fernández G. Hansen C. et al. (2019). Long-term impact of chronic variable stress in adolescence versus adulthood. *Prog. Neuropsychopharmacol. Biol. Psychiatry* 88 303–310. 10.1016/j.pnpbp.2018.08.003 30096330 PMC6165677

[B10] Cuesta S. Funes A. Pacchioni A. M. (2020). Social isolation in male rats during adolescence inhibits the Wnt/β-catenin pathway in the prefrontal cortex and enhances anxiety and cocaine-induced plasticity in adulthood. *Neurosci. Bull.* 36 611–624. 10.1007/s12264-020-00466-x 32078732 PMC7271097

[B11] Da Silva N. L. Ferreira V. M. M. De Padua Carobrez A. Morato G. S. (1996). Individual housing from rearing modifies the performance of young rats on the elevated plus-maze apparatus. *Physiol. Behav.* 60 1391–1396. 10.1016/S0031-9384(96)00254-5 8946480

[B12] Dalrymple-Alford J. C. Benton D. (1981). The effect of social isolation of the rat on open field activity and emergence. *Behav. Process.* 6 283–290. 10.1016/0376-6357(81)90007-3 24925818

[B13] Dimonte S. Sikora V. Bove M. Morgese M. G. Tucci P. Schiavone S. et al. (2023). Social isolation from early life induces anxiety-like behaviors in adult rats: Relation to neuroendocrine and neurochemical dysfunctions. *Biomed. Pharmacother.* 158:114181. 10.1016/j.biopha.2022.114181 36592494

[B14] Eiland L. Romeo R. D. (2013). Stress and the developing adolescent brain. *Neuroscience* 249 162–171. 10.1016/j.neuroscience.2012.10.048 23123920 PMC3601560

[B15] Ferdman N. Murmu R. Bock J. Braun K. Leshem M. (2007). Weaning age, social isolation, and gender, interact to determine adult explorative and social behavior, and dendritic and spine morphology in prefrontal cortex of rats. *Behav. Brain Res.* 180 174–182. 10.1016/j.bbr.2007.03.011 17477981

[B16] Figueiredo Cerqueira M. M. D. Castro M. M. L. Vieira A. A. Kurosawa J. A. A. Amaral Junior F. L. D. Siqueira Mendes F. D. C. C. D. et al. (2023). Comparative analysis between open field and elevated plus maze tests as a method for evaluating anxiety-like behavior in mice. *Heliyon* 9:14522. 10.1016/j.heliyon.2023.e14522 37025809 PMC10070366

[B17] Gloveli N. Simonnet J. Tang W. Concha-Miranda M. Maier E. Dvorzhak A. et al. (2023). Play and tickling responses map to the lateral columns of the rat periaqueductal gray. *Neuron* 111 3041–3052.e7. 10.1016/j.neuron.2023.06.018 37516112 PMC10552647

[B18] Hayashi H. Tateishi S. Inutsuka A. Maejima S. Hagiwara D. Sakuma Y. et al. (2025). Oxytocin facilitates human touch-induced play behavior in rats. *Curr. Biol.* 35 2916–2926.e3. 10.1016/j.cub.2025.05.034 40472853 PMC12201962

[B19] Hegberg N. J. Hayes J. P. Hayes S. M. (2019). Exercise intervention in PTSD: A narrative review and rationale for implementation. *Front. Psychiatry* 10:133. 10.3389/fpsyt.2019.00133 30949075 PMC6437073

[B20] Hol T. Van Den Berg C. L. Van Ree J. M. Spruijt B. M. (1999). Isolation during the play period in infancy decreases adult social interactions in rats. *Behav. Brain Res.* 100 91–97. 10.1016/S0166-4328(98)00116-8 10212056

[B21] Hori M. Hayashi T. Nakagawa Y. Sakamoto S. Urayama O. Murakami K. (2009). Positive emotion-specific changes in the gene expression profile of tickled rats. *Mol. Med. Rep.* 2, 157–161. 10.3892/mmr_00000077 21475806

[B22] Hori M. Shimoju R. Takunaga R. Ohkubo M. Miyabe S. Ohnishi J. et al. (2013a). Tickling increases dopamine release in the nucleus accumbens and 50 kHz ultrasonic vocalizations inin adolescent rats. *NeuroReport* 24 241–245. 10.1097/WNR.0b013e32835edbfa 23399995

[B23] Hori M. Yamada K. Ohnishi J. Sakamoto S. Furuie H. Murakami K. et al. (2014). Tickling during adolescence alters fear-related and cognitive behaviors in rats after prolonged isolation. *Physiol. Behav.* 131 62–67. 10.1016/j.physbeh.2014.04.008 24727339

[B24] Hori M. Yamada K. Ohnishi J. Sakamoto S. Takimoto-Ohnishi E. Miyabe S. et al. (2013b). Effects of repeated tickling on conditioned fear and hormonal responses in socially isolated rats. *Neurosci. Lett.* 536 85–89. 10.1016/j.neulet.2012.12.054 23313827

[B25] Ishiyama S. Brecht M. (2016). Neural correlates of ticklishness in the rat somatosensory cortex. *Science* 354, 757–760. 10.1126/science.aah5114 27846607

[B26] Kai L. M. Iglesias L. P. Kõiv K. Harro J. Wegener G. (2025). Ultrasonic vocalisations in the flinders sensitive line rat, a genetic animal model of depression. *Acta Neuropsychiatr.* 37:e3. 10.1017/neu.2024.61 39844375 PMC13130335

[B27] Knutson B. Burgdolf J. Panksepp J. (1998). Anticipation of play elicits high-frequency ultrasonic vocalizations in young rats. *J. Comp. Psychol.* 112 65–73. 10.1037/0735-7036.112.1.65 9528115

[B28] Komorowska J. Pisula W. (2003). Does changing levels of stress affect the characteristics of grooming behavior in rats? *Int. J. Comp. Psychol.* 16 237–246. 10.46867/IJCP.2003.16.04.01

[B29] Leussis M. P. Andersen S. L. (2008). Is adolescence a sensitive period for depression? Behavioral and neuroanatomical findings from a social stress model. *Synapse* 62 22–30. 10.1002/syn.20462 17957735

[B30] Lu C. Y. Liu D. X. Jiang H. Pan F. Ho C. S. H. Ho R. C. M. (2017). Effects of traumatic stress induced in the juvenile period on the expression of gamma-aminobutyric acid receptor type A subunits in adult rat brain. *Neural Plast.* 2017, 1–10. 10.1155/2017/5715816 28352479 PMC5352903

[B31] Lukkes J. L. Mokin M. V. Scholl J. L. Forster G. L. (2009). Adult rats exposed to early-life social isolation exhibit increased anxiety and conditioned fear behavior, and altered hormonal stress responses. *Horm. Behav.* 55 248–256. 10.1016/j.yhbeh.2008.10.014 19027017

[B32] Luo X.-M. Yuan S.-N. Guan X.-T. Xie X. Shao F. Wang W.-W. (2014). Juvenile stress affects anxiety-like behavior and limbic monoamines in adult rats. *Physiol. Behav.* 135 7–16. 10.1016/j.physbeh.2014.05.035 24907693

[B33] Lyttle K. Ohmura Y. Konno K. Yoshida T. Izumi T. Watanabe M. et al. (2015). Repeated fluvoxamine treatment recovers juvenile stress-induced morphological changes and depressive-like behavior in rats. *Brain Res.* 1616, 88–100. 10.1016/j.brainres.2015.04.058 25960352

[B34] Metts A. V. Yarrington J. S. Zinbarg R. Hammen C. Mineka S. Enders C. et al. (2023). Early-life adversity and risk for depression and anxiety: The role of interpersonal support. *Dev. Psychopathol.* 35 863–875. 10.1017/S0954579422000116 35285426

[B35] Mochizuki-Kawai H. Ichitani Y. Ayabe-Kanamura S. Yamada K. (2022). Odors associated with neonatal experiences with the dam have enhanced anxiolytic effects in rat. *Chem. Senses* 47:bjac019. 10.1093/chemse/bjac019 36056921

[B36] Moore N. L. T. Gauchan S. Genovese R. F. (2014). Adolescent traumatic stress experience results in less robust conditioned fear and post-extinction fear cue responses in adult rats. *Pharmacol. Biochem. Behav.* 120, 17–24. 10.1016/j.pbb.2014.01.011 24491436

[B37] Mora-Gallegos A. Fornaguera J. (2019). The effects of environmental enrichment and social isolation and their reversion on anxiety and fear conditioning. *Behav. Process.* 158 59–69. 10.1016/j.beproc.2018.10.022 30389595

[B38] Okada R. Fujiwara H. Mizuki D. Araki R. Yabe T. Matsumoto K. (2015). Involvement of dopaminergic and cholinergic systems in social isolation-induced deficits in social affiliation and conditional fear memory in mice. *Neuroscience* 299, 134–145. 10.1016/j.neuroscience.2015.04.064 25943484

[B39] Okada R. Matsumoto K. Tsushima R. Fujiwara H. Tsuneyama K. (2014). Social isolation stress-induced fear memory deficit is mediated by down-regulated neuro-signaling system and Egr-1 expression in the brain. *Neurochem. Res.* 39, 875–882. 10.1007/s11064-014-1283-5 24647971

[B40] Panksepp J. Burgdorf J. (2000). 50-kHz chirping (laughter?) in response to conditioned and unconditioned tickle-induced reward in rats: Effects of social housing and genetic variables. *Behav. Brain Res.* 115 25–38. 10.1016/S0166-4328(00)00238-2 10996405

[B41] Panksepp J. Siviy S. Normansell L. (1984). The psychobiology of play: Theoretical and methodological. *Neurosci. Biobehav. Rev.* 8 465–492. 10.1016/0149-7634(84)90005-8 6392950

[B42] Pinzón-Parra C. Vidal-Jiménez B. Camacho-Abrego I. Flores-Gómez A. A. Rodríguez-Moreno A. Flores G. (2019). Juvenile stress causes reduced locomotor behavior and dendritic spine density in the prefrontal cortex and basolateral amygdala in Sprague–Dawley rats. *Synapse* 73:e22066. 10.1002/syn.22066 30102793

[B43] Quan M. N. Tian Y. T. Xu K. H. Zhang T. Yang Z. (2010). Post weaning social isolation influences spatial cognition, prefrontal cortical synaptic plasticity and hippocampal potassium ion channels in Wistar rats. *Neuroscience* 169 214–222. 10.1016/j.neuroscience.2010.04.048 20438813

[B44] Ramirez S. Liu X. MacDonald C. J. Moffa A. Zhou J. Redondo R. L. et al. (2015). Activating positive memory engrams suppresses depression-like behaviour. *Nature* 522 335–339. 10.1038/nature14514 26085274 PMC5583720

[B45] Rudy J. W. Kuwagama K. Pugh C. R. (1999). Isolation reduces contextual but not auditory-cue fear conditioning: A role for endogenous opioids. *Behav. Neurosci.* 113, 316–323. 10.1037//0735-7044.113.2.316 10357456

[B46] Shimoju R. Shibata H. Hori M. Kurosawa M. (2020). Stroking stimulation of the skin elicits 50-kHz ultrasonic vocalizations in young adult rats. *J. Physiol. Sci.* 70:41. 10.1186/s12576-020-00770-1 32938369 PMC10717904

[B47] Simola N. Brudzynski S. M. (2018). Rat 50-kHz ultrasonic vocalizations as a tool in studying neurochemical mechanisms that regulate positive emotional states. *J. Neurosci. Methods* 310 33–44. 10.1016/j.jneumeth.2018.06.018 29959002

[B48] Song M. K. Lee J. H. Kim Y.-J. (2021). Effect of chronic handling and social isolation on emotion and cognition in adolescent rats. *Physiol. Behav.* 237 113440. 10.1016/j.physbeh.2021.113440 33940083

[B49] Tamura H. Miyazaki A. Kawamura T. Gotoh H. Yamamoto N. Narita M. (2024). Chronic ingestion of soy peptide supplementation reduces aggressive behavior and abnormal fear memory caused by juvenile social isolation. *Sci. Rep.* 14:11557. 10.1038/s41598-024-62534-w 38773352 PMC11109177

[B50] Toyoshima M. Yamada K. Sugita M. Ichitani Y. (2018). Social enrichment improves social recognition memory in male rats. *Anim. Cogn.* 21 345–351. 10.1007/s10071-018-1171-5 29488111

[B51] Vanderschuren L. J. M. J. Trezza V. eds (2014). What the laboratory rat has taught us about social play behavior: Role in behavioral development and neural mechanisms. Curr. Top. *Behav. Neurosci.* 16, 189–212. 10.1007/978-3-642-54913-7 24338663

[B52] Vargas J. Junco M. Gomez C. Lajud N. (2016). Early life stress increases metabolic risk, HPA axis reactivity, and depressive-like behavior when combined with postweaning social isolation in rats. *PLoS One* 11:e0162665. 10.1371/journal.pone.0162665 27611197 PMC5017766

[B53] Varty G. B. Paulus M. P. Braff D. L. Geyer M. A. (2000). Environmental enrichment and isolation rearing in the rat: Effects on locomotor behavior and startle response plasticity. *Biol. Psychiatry* 47 864–873. 10.1016/S0006-3223(99)00269-3 10807959

[B54] Whitten C. J. Kelly J. R. Gillespie A. L. Brooks H. J. B. Hooker M. K. Temple A. R. et al. (2025). From play date to stress fate: Juvenile social play rescues stress-induced changes in adult social behavior. *Dev. Psychobiol.* 67:e70020. 10.1002/dev.70020 39878574

[B55] Wright J. M. Gourdon J. C. Clarke P. B. S. (2010). Identification of multiple call categories within the rich repertoire of adult rat 50-kHz ultrasonic vocalizations: Effects of amphetamine and social context. *Psychopharmacology* 211 1–13. 10.1007/s00213-010-1859-y 20443111

[B56] Xie H. Shih C.-H. Aldoohan S. D. Wall J. T. Wang X. (2023). Hypothalamus volume mediates the association between adverse childhood experience and PTSD development after adulthood trauma. *Transl. Psychiatry* 13:274. 10.1038/s41398-023-02576-2 37542036 PMC10403516

[B57] Xu Y.-Y. Ge J.-F. Qin G. Peng Y.-N. Zhang C.-F. Liu X.-R. et al. (2015). Acute, but not chronic, stress increased the plasma concentration and hypothalamic mRNA expression of NUCB2/nesfatin-1 in rats. *Neuropeptides* 54 47–53. 10.1016/j.npep.2015.08.003 26297350

[B58] Yamamuro T. Senzaki K. Iwamoto S. Nakagawa Y. Hayashi T. Hori M. et al. (2010). Neurogenesis in the dentate gyrus of the rat hippocampus enhanced by tickling stimulation with positive emotion. *Neurosci. Res.* 68 285–289. 10.1016/j.neures.2010.09.001 20851720

[B59] Yee N. Schwarting R. K. W. Fuchs E. Wöhr M. (2012). Juvenile stress potentiates aversive 22-kHz ultrasonic vocalizations and freezing during auditory fear conditioning in adult male rats. *Stress* 15, 533–544. 10.3109/10253890.2011.646348 22150360

[B60] Zhao M. Wang W. Jiang Z. Zhu Z. Liu D. Pan F. (2020). Long-term effect of post-traumatic stress in adolescence on dendrite development and H3K9me2/BDNF Expression in male rat hippocampus and prefrontal cortex. *Front. Cell Dev. Biol.* 8:682. 10.3389/fcell.2020.00682 32850808 PMC7412801

[B61] Zolfaghari F. S. Pirri F. Gauvin E. Peeri M. Amiri S. (2021). Exercise and fluoxetine treatment during adolescence protect against early life stress-induced behavioral abnormalities in adult rats. *Pharmacol. Biochem. Behav.* 205:173190. 10.1016/j.pbb.2021.173190 33865889

