# Supplementary material for: Adolescent tickling incorporating handling is associated with reduced stress- and fear-related behaviors in socially isolated male rats
Source: Front Behav Neurosci. 2026 Sep 3;20:1869518. doi: 10.3389/fnbeh.2026.1869518 (PMC13602953; doi:10.3389/fnbeh.2026.1869518)
Supplement: Supplementary file 1 [file Table_1.docx]

Supplementary Material

**Supplementary Figure S1.** **Body Weight Changes from Baseline**

Body weight changes from baseline during up 3weeks to12 weeks. Data are shown as mean ± SEM.

**Supplementary Figure S2.** **Temporal changes in USV subtype call duration during repeated tickling stimulation**

(A) Upward ramp, (B) Step-down, and (C) Trill call duration during repeated tickling stimulation. Solid lines indicate juvenile foot-shock rats, and dashed lines indicate non-foot-shock rats. Data are presented as mean ± SEM.

**Supplementary Table S1.** **Temporal profile of USV subtype changes during repeated tickling stimulation**

Repeated-measures ANOVA showing the temporal profiles of 50-kHz USV subtype call counts and call duration during repeated tickling stimulation. Values represent *p* values for the main effects of Week and foot-shock and the Week × foot-shock interaction. Statistically significant (*p* < .05) and trend-level (*p* < .10) effects are shown in bold. † Greenhouse–Geisser-corrected results are reported when Mauchly's test indicated a violation of sphericity; otherwise, sphericity-assumed results are presented.

| Supplementary Table 1. Temporal profile of USV subtype changes during repeated tickling stimulation. | | | | | |  |
| --- | --- | --- | --- | --- | --- | --- |
|  |  |  |  |  |  |  |
| **USV subtype** | **Call counts** | | | **Call duration** | | |
|  | **Week** | **Foot-shock** | **Week × Foot-shock** | **Time** | **Foot-shock** | **Week × Foot-shock** |
| **Composite** | **0.01** | NS | NS | NS† | NS | NS† |
| **Upward ramp** | **0.007** | NS | **0.023** | NS† | NS | **0.033†** |
| **Downward ramp** | **< .001** | NS | NS | **0.002** | NS | NS |
| **Flat** | **0.003** | NS | NS | **0.003†** | NS | NS† |
| **Short** | NS | NS | NS | NS† | NS | NS† |
| **Multi-step** | NS† | NS | NS† | **0.001†** | NS | NS† |
| **Step up** | **0.089†** | NS | NS† | **0.089†** | NS | NS† |
| **Step-down** | **0.028†** | **0.044** | **0.026†** | **< .001†** | NS | **0.085†** |
| **Trill** | **0.014†** | NS | NS† | **0.004†** | **< .001** | **0.061†** |
| **Inverted U** | **0.081** | NS | NS | **0.047†** | NS | NS† |
|  |  |  |  |  |  |  |
